# Validation of Wired and Wireless Interconnected Body Sensor Networks

**DOI:** 10.3390/s19173697

**Published:** 2019-08-26

**Authors:** Anum Talpur, Faisal Karim Shaikh, Natasha Baloch, Emad Felemban, Abdelmajid Khelil, Muhammad Mahtab Alam

**Affiliations:** 1 Department of Telecommunication Engineering, Mehran University of Engineering and Technology, Jamshoro 76062, Pakistan; 2 Computer Engineering Department, Umm Al-Qura University, Makkah 21514, Saudi Arabia; 3 Department of Computer Science, Landshut University of Applied Sciences, Landshut 84036, Germany; 4 Thomas Johann Departmnt of Electronics, Tallinn University of Technology, Tallinn 19086, Estonia

**Keywords:** body sensor networks, wireless body area network, topology/power control, e-health system

## Abstract

Current medical facilities usually lead to a very high cost especially for developing countries, rural areas and mass casualty incidents. Therefore, advanced electronic health systems are gaining momentum. In this paper, we first compared our novel off the shelf experimental wired Body Sensor Networks (BSN), that is, Digital First Aid (DigiAID) with the existing commercial product called as Hexoskin. We showed the viability of DigiAID through extensive real measurements during daily activities by both male and females. It was found that the major hurdle was wires to be worn by the subjects. Accordingly, we proposed and characterized the wireless DigiAID platform for wireless BSN (WBSN). Understanding the effect of body movements on wireless data transmission in WBSN is also of major importance. Therefore, this paper comprehensively evaluates and analyzes the impact of body movements, (a) to ensure transmission of data at different radio power levels and (b) its impact on the topology of the WBSN. Based on this we have proposed a dynamic power control algorithm that adapts the transmitting power according to the packet reception in an energy efficient manner. The results show that we have achieved substantial power savings at various nodes attached to the human body.

## 1. Introduction

Electronic health (e-Health) and mobile health (m-Health) are increasingly considered to be key drivers for the progress of Internet of Things (IoT) based tele-health systems [1]. The current social, economic and ecological trends of massive urbanization and climate changes impose painful changes to the healthcare landscape and further complicate the e-Health paradigm. The massive urbanization moves the focus of healthcare to urban areas and therefore hardens the healthcare capabilities in rural areas. Similar problems are also seen in developing countries where even in urban areas the proper healthcare facilities are not available.

**Figure 1:**
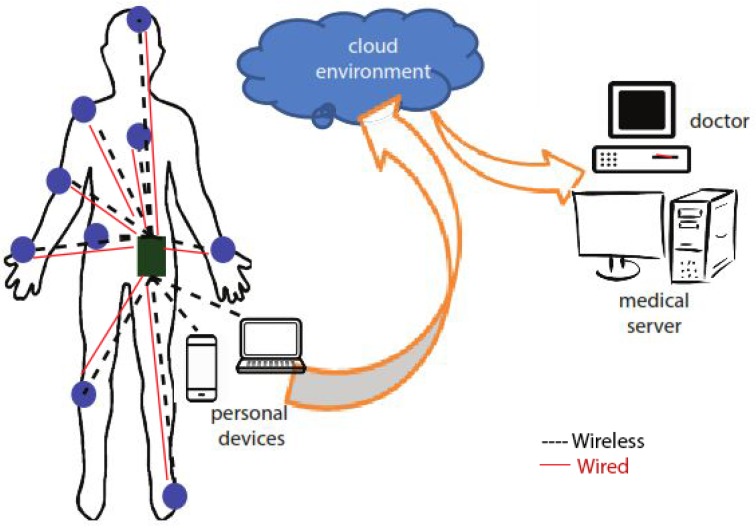
Generic body sensor network scenario.

Fortunately, advances have been made towards smart living and health monitoring systems that support tele-medicine. In particular, Body Sensor Networks (BSN) [2] and wireless communications [3] represent powerful techniques for significantly advance healthcare in developing countries. In BSN, wearable sensor nodes are deployed over a human body. The sensors can be used externally on the body or inside the human body to measure certain health parameters. For example, measuring the heart rate, body temperature or recording a prolonged electrocardiogram (ECG). These wearable or implantable sensors communicate with the base station (BS) to collect the data and then transmit it to the medical server or personal device (smart phones, laptop) or the doctor for further assessment/treatment. BSN is mostly used for long time health monitoring. One of the advantages of using this technology is that a patient need not to stay longer in hospitals and can be monitored remotely. BSN can be either wired or wireless in nature. In wired BSN the sensors scattered on the body are connected using wires to BS. However, in wireless BSN (WBSN) the sensors communicate wirelessly to BS. The generic wired and wireless BSN scenarios are shown in Figure 1.

In Reference [4] we proposed a wired BSN called as Digital First Aid (DigiAID), that can be applied to patients in emergency situations to compute the level of the urgency in real time and update the corresponding hospital prior to arrival of patients. The DigiAID can also be utilized by normal people to monitor and provide early health indicators to avoid dangerous health situations. In order to validate our proposed design, we have compared the DigiAID system with the existing commercially available product called as Hexoskin [5] by extensive experiments both on male and female subjects. The wired DigiAID collects three vital parameters for health monitoring, that is, heart rate (HR), respiratory rate (RR) and mental status (MS) using three sensors. The sensors are paced on finger, near nose and in hand. Various other sensors can also be integrated in the proposed design depending on the application requirement. However, the wired nature of the design restrict the amount of sensors and its placement on the body. Accordingly, we developed a wireless DigiAID platform to overcome the problems of wired platform. We have used eight sensor nodes over human body at different places pertaining to monitoring application of different body parameters [6] for our wireless DigiAID. The increased number of nodes is also intended to scrutinize the behavior of wireless system in a manifold structure. We converted our wired DigiAID system wirelessly using ZigBee standard for transmitting data over Radio Frequency (RF). ZigBee/IEEE 802.15.4 is adopted by most of the health care networks due to its inherent features of low power consumption [6,7,8]. It has ben observed that body movements and postures can have significant effect on the efficient data delivery [9]. Constant motion of human body can alter the position of sensor nodes. These sudden changes can drop links of the nodes with the BS which may lead to no data transfer between the sensors and BS. This results in unreliable delivery of data which can be life threatening for a patient. Another key contribution of the paper is to perform series of experiments to observe the impact of body movements over the topology and power utilized to transfer the data in wireless DigiAID. Power efficiency is the most important factor for WBSN because of the fact that the sensor nodes are not only smaller in size but also have limited battery life [10,11]. Different techniques for reducing the power levels have been discussed in References [12,13,14,15,16,17,18,19,20,21,22,23] for WBSN. The goal is to keep a low radio power level for transmission and reception which is not only safe for human body, efficient for network protocols and also save battery life. In this paper, to ensure efficient power consumption, a dynamic power control algorithm is proposed which depends upon Received Signal Strength Indicator (RSSI) values. Main characteristics of proposed strategy is to observe the effect of different radio power level at regular body movements for communication reliability. A dynamic power control mechanism is considered, where three different transmit radio levels are taken into account. This adaptive strategy works depending on the average Packet Reception Ratio (PRR). Beacon packet fields are used to trigger the transmitter for a change in radio level to ensure the reliable transfer of data.

Once the data is gathered by BS it will be transmitted onwards to a server or cloud. Generally, the amount of data transmitted by BSN/WBSNs is enormous. The collected data at cloud can be used for further analysis and predictions about health routines and the spread of diseases [24,25]. Recently, deep architectures [26,27], machine learning [28,29,30], signal processing [31] and data fusion [32,33] are utilized on the gathered data to understand the activities performed by the subjects.

The rest of the paper is organized as follows. Section 2 describes the wired and wireless BSN design. Section 3 discusses about the proposed energy efficient data transfer mechanism for wireless BSN design. Experimental setup is explained for wired and wireless systems in Section 4. The performance evaluation is carried in Section 5. Finally, Section 6 concludes and provides future direction.

## 2. BSN System Design

In this section we describe the wired and wireless design of proposed BSN. The major difference between wired and wireless BSN is that in wired BSN the sensor on body are connected to BS using wires. However, in wireless BSN the sensors are connected wirelessly to BS.

### 2.1. DigiAID—A Wired BSN

Inspired by first aid box, our innovation is to build a set of new digital first aid tools using BSNs for health monitoring using reasonable cost sensors (Electrocardiogram (ECG), Phonocardiogram, Esophagogastroduodenoscopy, pressure, pulse, temperature and oximeter etc.).

**Figure 2:**
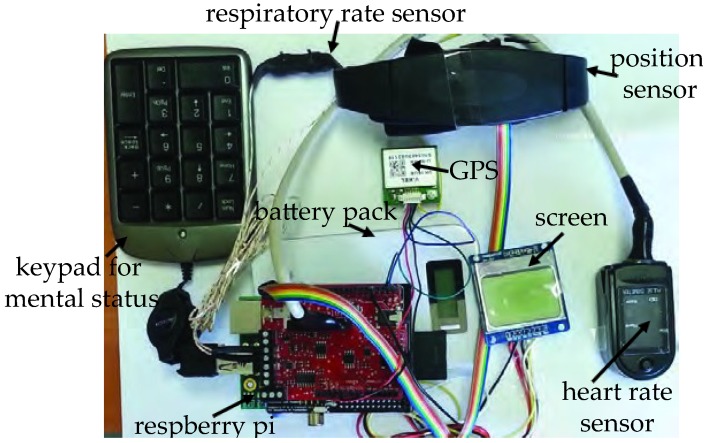
DigiAID hardware components.

DigiAID [4] is a tool that we proposed to bring rapid assistance to individuals in emergency cases. The rapidity is ensured through an early diagnosis and assessment of the health condition without an expert in loop. All what we require is a BSN that can measure important health indications in real-time and then be able to judge on the criticality of an individual. Through this platform, we continuously monitor three elementary critical vital signs, that is, RR, HR and MS. For the implementation of DigiAID, we selected the eHealth Sensor Shield [34]. Being open-source hardware/software and low-cost, the platform promises a faster acceptance and spread of our implementation, as well as large-scale trials in daily life activities. There are primarily three sensors attached to the DigiAID, that is, airflow sensor, pulse oximeter and position sensor. The airflow sensor is used to check RR of the patient. The pulse oximeter is a noninvasive method of indicating the arterial oxygen saturation of functional hemoglobin. It is very useful in any setting where the patient’s oxygenation is unstable. Alongside it also provides RR of the patient. The position sensor consists of three axis accelerometer to record the body position of the patient such as standing, sitting, supine, prone, left and right. These sensors are used to help an individual as well as on-site paramedic staff and remote doctors in understanding the patient health metrics and classifying the patients that have serious illness. The useful metrics are displayed on the display along with Global Positioning System (GPS) coordinates for patient location. DigiAID has a keypad interface for the patient to type and show his consciousness, that is, MS regarding the sensory perception. The value to be inputted by the patient is displayed on the screen. We primarily plan to use a microphone along with a 3D accelerometer to detect the MS of the patient. However, the automation of the MS capture is an ongoing step. It should be noted that DigiAID can be easily used to monitor health parameters during routine activities and is not restricted only to emergency situations. The complete hardware solution is shown in Figure 2.

Several experimental BSN platforms exist such as References [35,36,37]. Only a subset of them enable biometric and medical applications [34,38,39,40,41,42]. The core functionality provided by these platforms is to monitor the human body in real time by capturing some of its key health state indicators and to collect sensitive data for subsequent analysis and medical diagnosis.

Several academic platforms such as References [39,40,43], MobiHealth [44,45], MYOTEL [42], Personal Health Monitor (PHM) [41,46] have been developed and applied in various clinical settings. However, these platforms are still unavailable commercially and therefore cannot be used to compare with DigiAID. On the other hand, more and more commercial platforms are being released. Examples include the Shimmer Health BSN [38,47] from Intel, the Simband health sensor platform [48] from Samsung and biometric smart shirt by Hexoskin [5]. From above systems we have chosen Hexoskin because its system design is very close to DigiAID and can be easily compared due to its portability and providing similar health indicators.

### 2.2. Wireless DigiAID—A Wireless BSN

In wireless DigiAID, the sensors transmit data wirelessly to BS compared to wired transmission in DigiAID system. This part of research briefs the challenges faced by DigiAID, when it is converted into wireless to increase the flexibility of system and usage in routine life. We highlight the problems related to the deployment of reliable and energy efficient wireless DigiAID System. Collecting and transmitting information to and from the nodes with reliability and efficiency is a challenging task. The effects of data transmission with respect to three regular body movements (i.e., Walking, Sitting and Sleeping) are observed in closed room environment. After successful deployment, the sensor nodes are configured in such a way to investigate how power would be controlled for different body movements. Since, prolonged lifetime of the network is a major design goal for WBSNs, due to the limitation in battery resources. About 80% of the energy is consumed in communication purpose [49]. Therefore, an energy efficient data transmission algorithm plays a key role to save the energy and prolong the network lifetime. Accordingly, we have proposed an algorithm to limit the use of energy, while providing a high communication reliability by utilizing adaptive power controlling mechanism for wireless DigiAID system. The proposed algorithm avoids the loss of packets due to irregular body movements.

**Figure 3:**
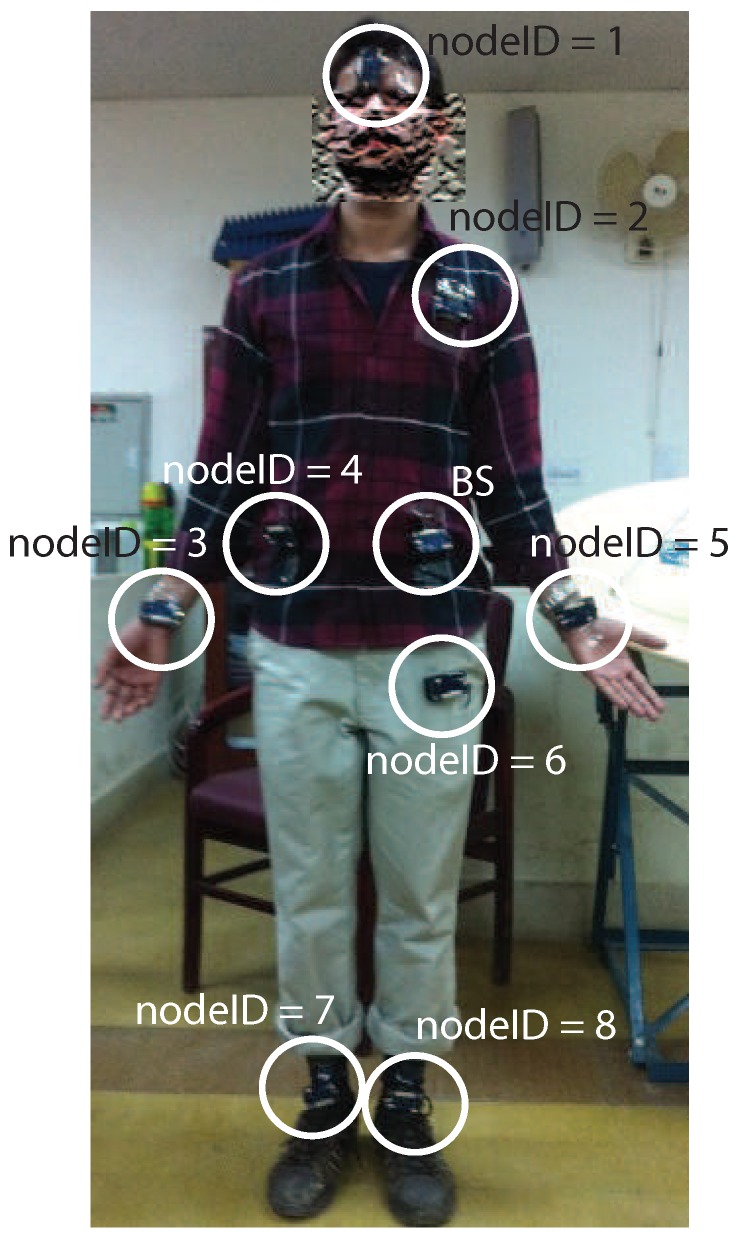
Wireless DigiAID system.

In wireless DigiAID, we used eight sensor nodes placed on the entire body of the subject. The sensor nodes are placed on head, shoulder, back, thigh, two hands and two ankles. The BS is located at the belly of the subject and collect the data from all other sensor nodes. We considered IRIS motes [50] to represent the sensor nodes positioned at different locations for measuring physiological parameters over the human body as shown in Figure 3.

## 3. Proposed Energy Efficient Data Transfer in Wireless DigiAID

Network topology plays an important role for efficient and reliable data transfer. In wireless DigiAID the topology is dynamically changing due to body movements and postures. In order to cope with dynamically changing topology we have used Collection Tree Protocol (CTP) [51], a default routing protocol in TinyOS [52]. CTP has a capability to efficiently switch to second route in case the first fails, with almost 90% reliability of packet transmission. Therefore, to examine topological effects for wireless DigiAID, all motes are programmed with CTP using TinyOS. Each node is labeled with unique Identification (ID) from 1 to 8 respectively and the BS is assigned with ID = 0. All the readings are taken for an estimated time of 10 to 15 min. The transmission power is managed by the IRIS mote radio. It provides 16 transmission levels ranging from +3 dBm to −17.2 dBm. The radio power is tuned accordingly to check the reliability of packets and to monitor the change in topology for each body movement (sitting, walking and sleeping). In order to estimate the packet reception, data is transmitted and allowed to form different topology for each power level. The data rate of each sensor node is one packet per second. For HR and RR, the frequency of data is low from the sensor. Thus, the loss of data is minimal and fulfil the requirement for proof of concept. However, for more sophisticated health parameters such as ECG or EEG, where the frequency of data from sensors is high the data can be aggregated [53,54] and/or the data rate can be increased [55,56]. For retrieving data from multiple sensors we adapted random timers to transmit the data. For higher data rates various MAC protocols for WBAN can be considered to avoid collisions [57,58]. The readings are taken at four different transmission (TX) power levels, that is, *TX*_1_ = 3.0 dBm (maximum power of IRIS mote), *TX*_2_ = −2.2 dBm, *TX*_3_ = −17.2 dBm (minimum power of IRIS mote) and *TX*_4_ = −17.2 dBm (the antenna is covered with aluminum foil). The aluminum foil is used to further degrade the channel beyond the minimum power of IRIS mote to observe its impact on the communication. For each body movement four experiments have been performed to observe the effect of the power on the topology formed.

According to Reference [59], the total radio energy consumed during data transmission is given as follows

$$E_{TX}\left(k,d,n\right)=E_{TX_{el}}*k+E_{a}\left(n\right)*k*d^{n}$$

where, *k* is the number of bits transmitted over distance *d*, with the path loss coefficient *n*. $E_{TX_{el}}$ and $E_{a}$ are the transmitter energy dissipated by its circuitry and amplifier respectively. By keeping all parameters of above equation constant, except *d* and *n*, the equation becomes,

$$E_{TX}\propto d^{n}$$

This elucidation says greater node separation and greater path loss exponent results in a greater expenditure of energy during transmission. This energy wastage is due to the increase of path loss with the increasing distance. To estimate the path loss with the increasing distance for a wireless BSN, from Reference [60] it is given as,

$$PL_{dB}=PL_{0,dB}+10*n*log\left(d/d_{o}\right)$$

**Figure 4:**
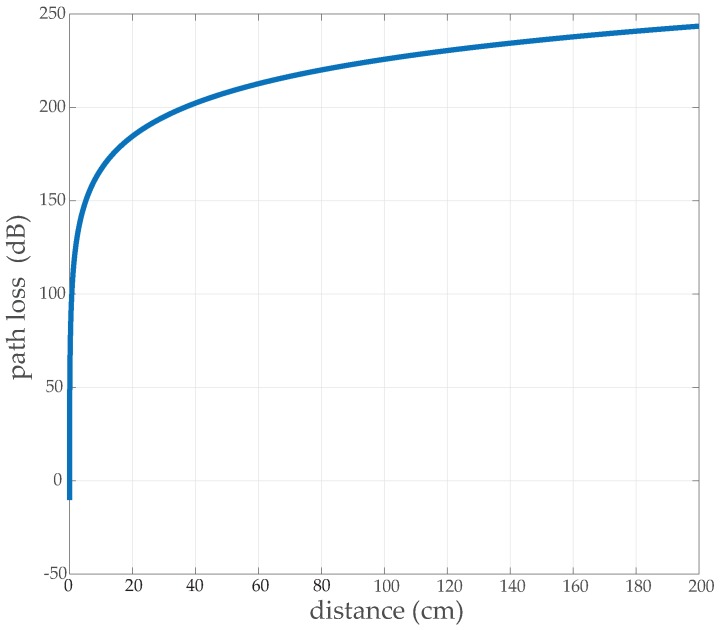
Path Loss for typical wireless body sensor network (WBSN) environment.

The *PL_dB_* gives us path loss at distance *d* while considering the reference path loss *PL*_0,*dB*_ at a reference distance of *d_o_*. For WBSN environment, from Reference [61] *n* = 5.9 and *PL*_0,*dB*_ = 48.8 dB at *d_o_* = 10 cm. The relation of path loss corresponding to increasing distance would be given as in Figure 4. Here, the Non-Line-Of-Sight (NLOS) communication is considered due to the obstructed irregular surface of the human body.

**Figure 5:**
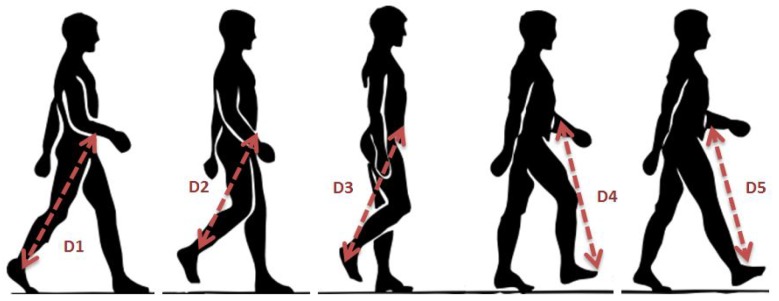
Distance variation between transmission node and base station while walking.

Considering the movement of walking as shown in Figure 5, if we consider the node of ankle, it can be shown that with every step a person is taking, distance between sending node and BS node is continuously changing. Sometimes minimum power is enough to send our data reliably or sometimes it may result in loss of data. This loss will be due to increasing distance and path loss. In order to make transmission, free of loss, adequate level of radio transmission is important to maintain the increasing distance.

The proposed algorithm avoids wastage of the expensive battery resources while maintaining a reliable communication using following steps. Initially, all sending nodes will send data packets at lowest transmission power level *TX_low_*. With each data packet, its respective RSSI is also received. The PRR of each sending node is calculated in a proposed algorithm to analyze the link quality. At every reception, previous value of *PRR_n_*[*t* − 1] is overwritten with new *PRR_n_*[*t*] through taking the average. After every estimated time *t_est_* seconds, PRR is checked. Different PRR threshold will be defined for different health parameters depending on the sensitivity of data. For PRR greater than threshold, TX level is maintained same (trigger bits = 10). And if PRR is consistently greater than threshold then TX level will be decreased (trigger bits = 01) to save energy. For PRR less than threshold, TX level is increased (trigger bits = 00) from *TX_low_* to *TX_medium_*. It is important to increase the TX level in order to overcome the loss and send data reliably to the BS. If PRR is good with *TX_medium_* then it remains at same level. Otherwise, again TX level will rise to even greater value (i.e., herewith given experimental setup, it will become *TX_high_*). The trigger for increment and decrement of power levels is given to TX from BS via beacon packets. In the given scenario, three power levels are used. This range of power levels will vary with different experimental systems. The time *t_est_* is different for each node depending upon its priority. The priority level varies depending upon the type of medical data transmitted by that node. Here in our experiment, we have kept *t_est_* constant (i.e., *t_est_* = 10 s) since the algorithm is analyzed here using dummy data. A 2-bit field (i.e., trigger bits) of beacon message is used in a given algorithm to trigger sending node for choosing appropriate TX power. Algorithms 1 and 2 will run at sending node and BS respectively. 

**Algorithm 1:** At Sending Node.
Set TX = *TX_low_* %*Start transmission with lowest power*After every *t_est_* seconds, check for 2-bit trigger field of beacon message***if*** trigger bits = 00 ***then***TX is increased with step size of *xyz*; exit();***elseif*** trigger bits = 01 ***then***TX is decreased with step size of *xyz*; exit();***elseif*** trigger bits = 10 ***then***TX is same; exit();
***end***



**Algorithm 2:** At BS.
calculate *RSSI_n_* for each *nth* nodecalculate *RX-Power_n_* from *RSSI_n_* % Received Power is analyzed for each node.calculate *PRR_n_* for all nodes***while*** transmission is ***ON***:***if**PRR_n_* > *Threshold_n_* ***then*** % Threshold is different for each node (sensitivity dependent)trigger bits = 10; exit();***elseif**PRR_n_* < *Threshold_n_* ***then***trigger bits = 00; exit();***elseif**PRR_n_* is continuously > *Threshold_n_* ***then***trigger bits = 01; exit();
***end***
***for*** (each *t_sec_* fired) ***do***Replace *PRR_n_* with avg(*PRR_n_*[*t* − 1], *PRR_n_*[*t*]) % Replace every new PRR with the average of new PRR + previous PRR
***end***



## 4. Experimental Setup

**Figure 6:**
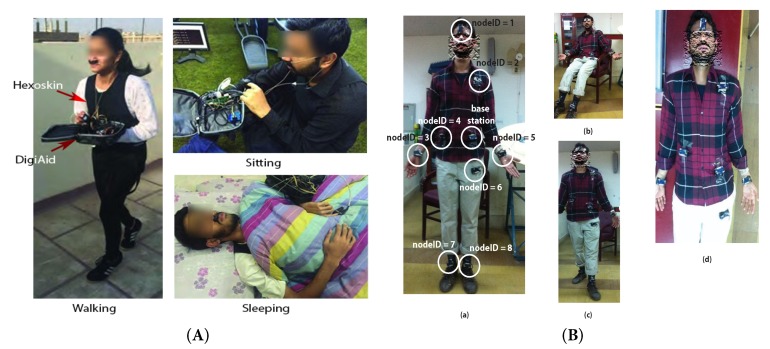
Positioning and movements of different subjects during experiments. (**A**) Wired DigiAID and Hexoskin; (**B**) Wireless DigiAID.

In order to compare DigiAID and Hexoskin, we consider the daily activities (i.e., sitting, walking and sleeping) of a healthy individual. For experiments the DigiAID with pulse-oximeter and airflow sensor, are placed on the human body to sense the physiological parameters, that is, HR and RR. The third parameter, that is, consciousness or MS is assumed to remains true during these activities. During experiments the subject also wears Hexoskin along with wired DigiAID to get the readings from both the platforms simultaneously as shown in Figure 6A. For each activity the person wears the DigiAID and Hexoskin for 30 min. During this period relevant parameter values are logged. According to Reference [62] the normal ranges for respiratory and heart rate at different positions is shown in Table 1.

The experiments were conducted by 5 males and 5 females from the age group of 18–25 years. The collected values from the subjects were averaged for the analysis. The values from DigiAID were collected from the system, whereas the values of Hexoskin were stored over the cloud and were downloaded from the online Hexoskin account.

For wireless DigiAID experiments (as shown in Figure 6B) we used nine nodes for 30 min and every second the data is transmitted to BS with some delay. Similarly, for wireless DigiAID 5 male and 5 female subjects from the age group of 18–25 were selected and the collected data is averaged for the analysis.

## 5. Performance Evaluation

### 5.1. Comparing DigiAID and Hexoskin

**Figure 7:**
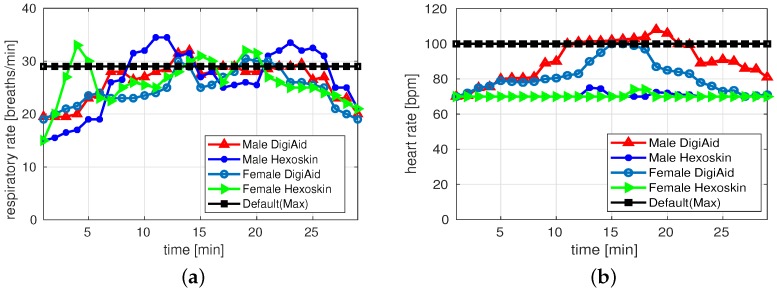
Measurements during walking activity. (**a**) Respiratory rate; (**b**) Heart rate.

Figure 7a,b depict results of RR and HR for walking activity of both male and female subjects. Generally, both platforms captured the results of RR within the range. Exceptionally at few instances the Hexoskin detected higher RR than the specified range. For both male and female DigiAID and Hexoskin performs equally good to collect RR values. For HR, Hexoskin values are again stable and provide values around 70 beats/min. Whereas, the values from DigiAID are deviating and difference between both platforms is huge at certain intervals revealing that pulse-oximeter may not be the good sensor choice to be utilized.

**Figure 8:**
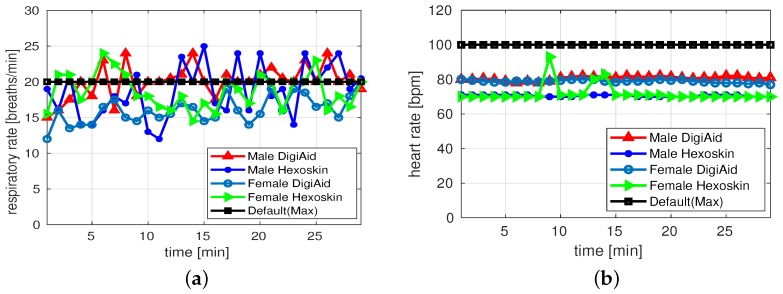
Measurements during sitting activity. (**a**) Respiratory rate; (**b**) Heart rate.

Figure 8a,b show the RR and HR of the both the male and female subjects during sitting scenario. The captured RR for females is within range for both DigiAID and Hexoskin, whereas for males both systems have captured slightly higher values. In both male and females, we observe that the behavior of Hexoskin is more fluctuating compared to DigiAID mainly due to usage of stretch based sensor in Hexoskin than air flow based sensor in DigiAID. The logged values of HR show that both platforms have captured values which are within range. We observe that HR values of Hexoskin are more stable than DigiAID. This is mainly because DigiAID take HR values using pulse-oximeter sensor compared to single channel ECG sensor used by the Hexoskin, which captures more accurate results. However, the results captured by DigiAID are also acceptable.

**Figure 9:**
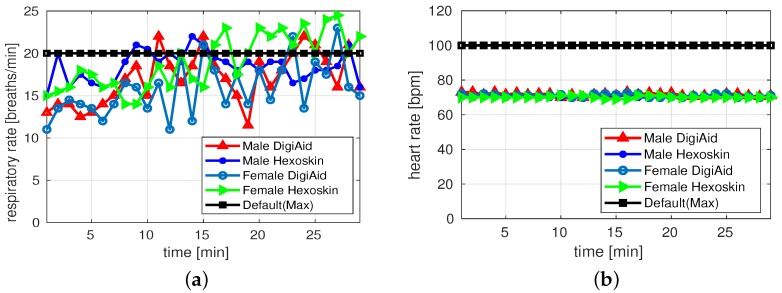
Measurements during sleeping activity. (**a**) Respiratory rate; (**b**) Heart rate.

Figure 9a shows results of RR for sleeping activity. We observe that for both platforms there are fluctuations in reading. However, the results for both male and female are within range except in few cases where Hexoskin values are beyond the range. Figure 9b depicts the HR results for sleeping activity. Both DigiAID and Hexoskin values are smooth and shows no fluctuations. In this scenario both sensors are performing well for male and female subjects.

Table 2 highlights the comparison between DigiAID and Hexoskin and summarizes our analysis for different activities. Here *good* represents the condition where majority of values are within allowable range as shown in Table 1, *satisfactory* where some deviation is observed and *not satisfactory* where majority of values are not in range. During walking situation, DigiAID is highly accurate for RR as well as HR measurements. During sitting position, DigiAID is highly accurate for RR whereas results are not satisfactory for HR. However, DigiAID is again accurate for RR and HR in sleeping posture. Generally, DigiAID promises to be fundamental tools to optimize the rescue operations by reducing the diagnosis time, which may significantly impact the success rate of saving life, especially in areas of low medical coverage such as rural or mass casualty incident areas. Furthermore, it is observed that DigiAID performed equally good to commercial product in all activities. It was also revealed from the subjects performing experiments that the wired system was major hurdle in smoothly performing the experiments. This leads us to experiment a wireless DigiAID design solution.

### 5.2. Wireless DigiAID

This section investigates the results achieved from wireless DigiAID design. The analyses of collected results provide an in-depth description of the packet reception at different power levels when a human body is performing routine movements. Here, the number of nodes is increased to nine for the application of other health parameters and dummy data is transmitted by each sensor node for the purpose of research. The increase in number of nodes is also intended to scrutinize the behavior of wireless system in a manifold structure. The evaluation of power results is performed using MATLAB with log data generated from IRIS motes. It proves *TX*_1_ as sufficient transmit power level to provide good enough packet reception. But the usage of *TX*_1_ means high energy consumption. Therefore, dynamic control based algorithm is simulated to provide a good percent of PRR with the prevention of energy wastage. The simulation results provide the analyses of proposed dynamic control based algorithm with an in-depth description of the energy saving at different power levels when a human body is performing routine movements. The behavior of wireless DigiAID methodology can further be investigated by evaluating the below obtained results.

**Figure 10:**
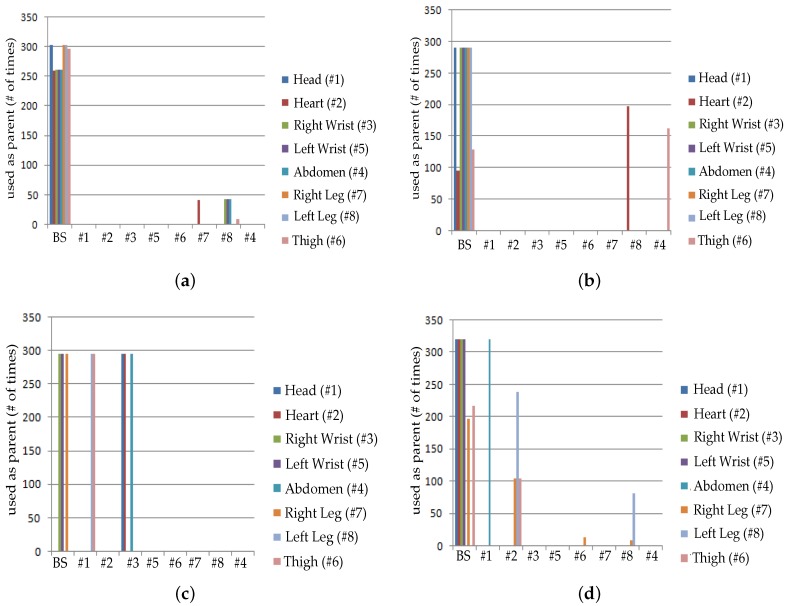
No. of hops while walking activity. (**a**) At *TX*_1_; (**b**) At *TX*_2_; (**c**) At *TX*_3_; (**d**) At *TX*_4_.

**Figure 11:**
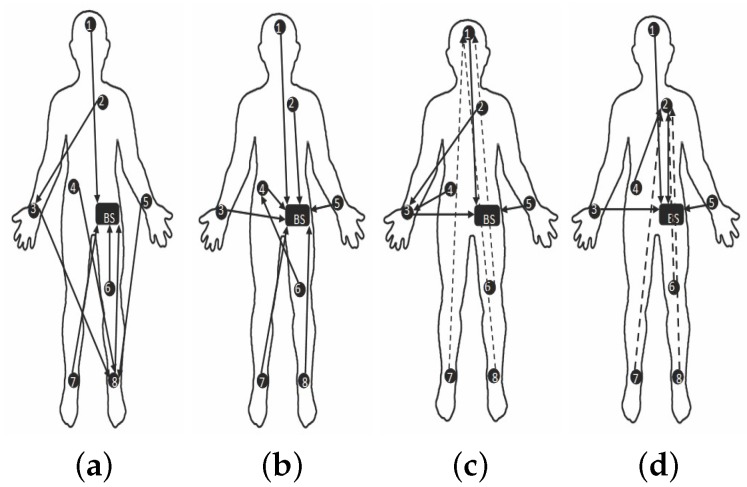
Typical network topology while walking activity. (**a**) At *TX*_1_; (**b**) At *TX*_2_; (**c**) At *TX*_3_; (**d**) At *TX*_4_.

Figure 10 and Figure 11 are captured when a person is walking back and forth in a room for a time interval of 10 min. For maximum transmit power (Figure 11a), initially single-hop topology is formed but due to frequent motion some node follows multiple hops to reach BS or sink. This multi-hop behavior of node 2, 3, 4 and 5 has used other nodes as their parents as shown in Figure 10a.

Similarly, at medium transmit power (Figure 11b), node maintains single-hop toward sink for around 5 min and after 5 min, node 6 is following multi-hop behavior whereas all the remaining nodes are continuing single-hop transmission. The node 2 has also used multi-hop behavior and used a parent as shown in Figure 10b. After sometime, it again maintains single-hop connection toward sink. Figure 11c,d shows that due to minimum transmit power and trivial body movements of walking, from the very start most of the nodes are following multi-hop to reach the BS. Even some nodes maintain multihop behavior for all time as shown in Figure 10c,d.

**Figure 12:**
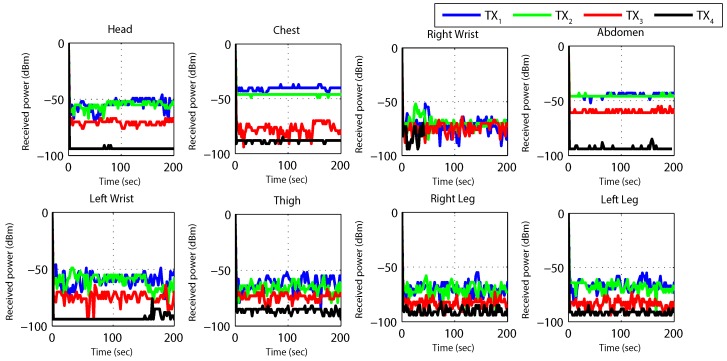
RX power based on default TX power while walking activity.

**Figure 13:**
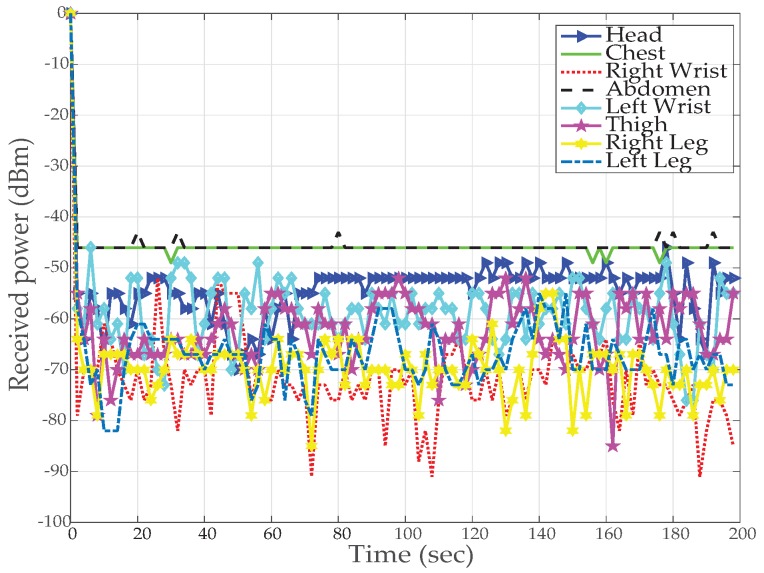
RX power using proposed dynamic control algorithm while walking activity.

Figure 12 and Figure 13 results are captured when a person is walking back and forth in a room for a time interval of 3–4 min. Figure 12 shows received power against time at BS calculated using RSSI values for different nodes. For the fixed maximum transmit power of 3 dBm graph shows that the received power is widely fluctuating around average values of −40 dBm to −80 dBm. Nodes at the wrists, thigh and legs show poor performance (received power is always less than −55 dBm) as compared to nodes at the chest, abdomen and head (received power is always greater than −55 dBm) due to movement while walking. The overall performance of the received power is very good for high transmit power and no loss in total number of received packets is observed.

**Figure 14:**
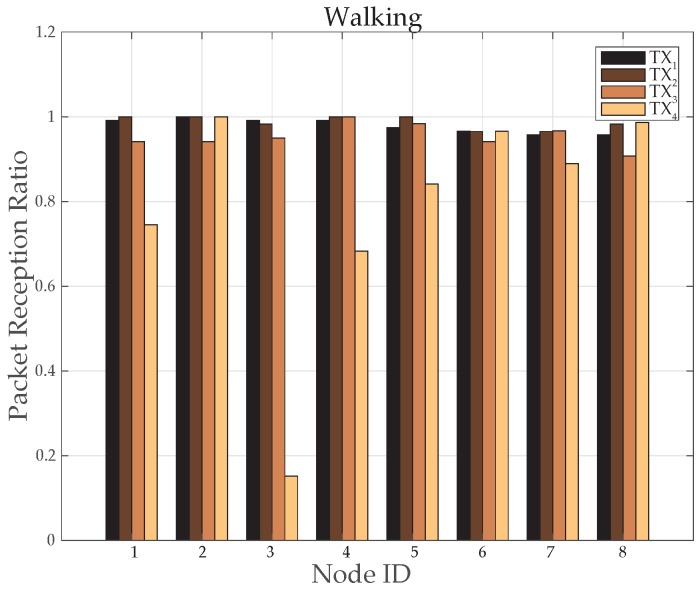
Packet Reception Ratio at various nodes while walking activity.

The results for *TX*_2_ show the variation between −50 dBm to −80 dBm with zero percent packet loss. This proves medium power to be more efficient for the wireless DigiAID network as compared to earlier case. Further evaluation of minimum transmits power (*TX*_3_) shows the deviation between −70 dBm to −90 dBm. *TX*_4_ power level results depicts that even lesser power can be used for transmission of data packets (with losses) which would be helpful in escalating the battery life time. However, at the same time it provides very poor PRR (Figure 14), whereas *TX*_1_ to *TX*_2_ is showing a very good level of packet reception. Therefore, with adopting dynamic control based transmission there is always a use of *TX*_1_ or *TX*_2_ to provide a good level of received power (Figure 13) with the energy efficiency. This also shows a tradeoff between low power and data loss, if the patient’s data is of great importance than extreme low power can never be advisable.

**Figure 15:**
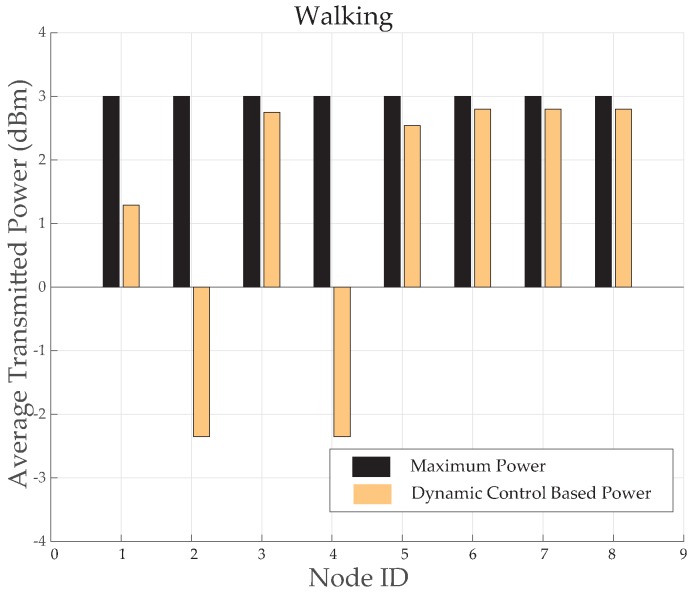
Average TX power consumption while walking activity.

Figure 15 provides a comparison of average transmitted power consumed before and after implementing the dynamic control based algorithm. Compared to maximum transmission power of IRIS mote, the node number 3, 5, 6, 7 and 8 has saved 3% to 8% of power. However, node 1 has saved 29% of power and node 2 and 4 has saved upto 90% of power. It proves this algorithm more energy efficient for routine body movement based communication.

**Figure 16:**
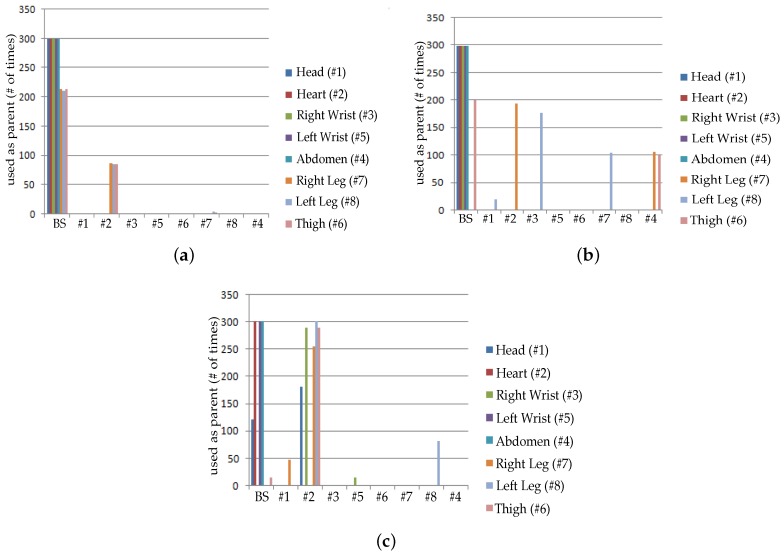
No. of hops while sitting activity. (**a**) At *TX*_2_; (**b**) At *TX*_3_; (**c**) At *TX*_4_.

**Figure 17:**
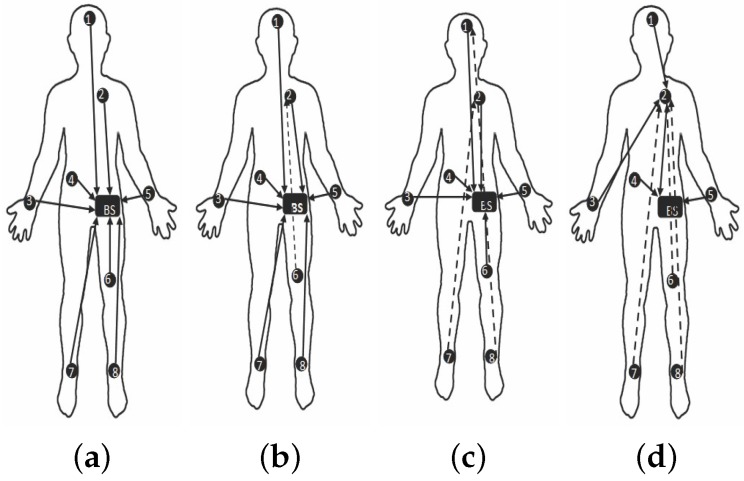
Typical network topology while sitting activity. (**a**) At *TX*_1_; (**b**) At *TX*_2_; (**c**) At *TX*_3_; (**d**) At *TX*_4_.

Figure 16 and Figure 17 are showing results for sitting postures, maintained for 10 min. In contrast to walking, topological behavior maintained while sitting at *TX*_1_ (Figure 17a) and *TX*_2_ (Figure 17b) is less complex because of the fact that body movements are not that frequent. At *TX*_2_ only 3 nodes have used a parent for few minutes. Whereas, all other nodes maintain direct connection towards sink which can be seen from Figure 16a. The main difference is observed for *TX*_3_ power level (Figure 17c) where more number of nodes has used other nodes as their parent to reach sink as estimated from greater number of nodes toward other nodes shown in Figure 16b.

In particular, it is observed that node 6 change its behavior only once whereas the behavior of node 7 and 8 vary 3 times for successful packet transmission. At *TX*_4_ (Figure 16c and Figure 17d), shows that again and again large number of nodes are changing their parents and only three nodes are making direct connection whereas all the remaining nodes have chosen a multi-hop route.

**Figure 18:**
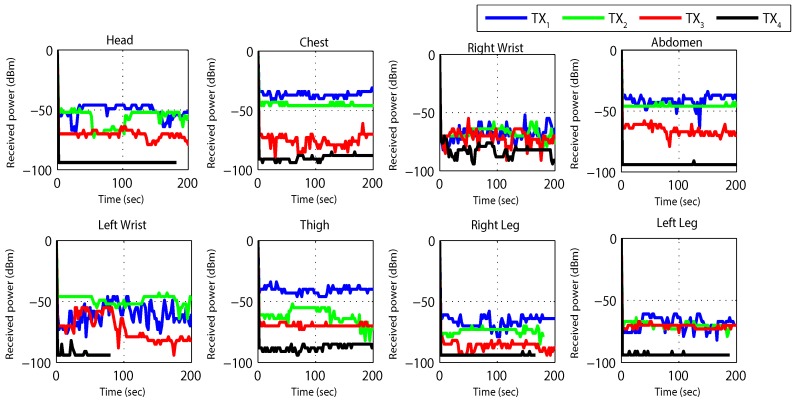
RX power based on default TX power while sitting activity.

**Figure 19:**
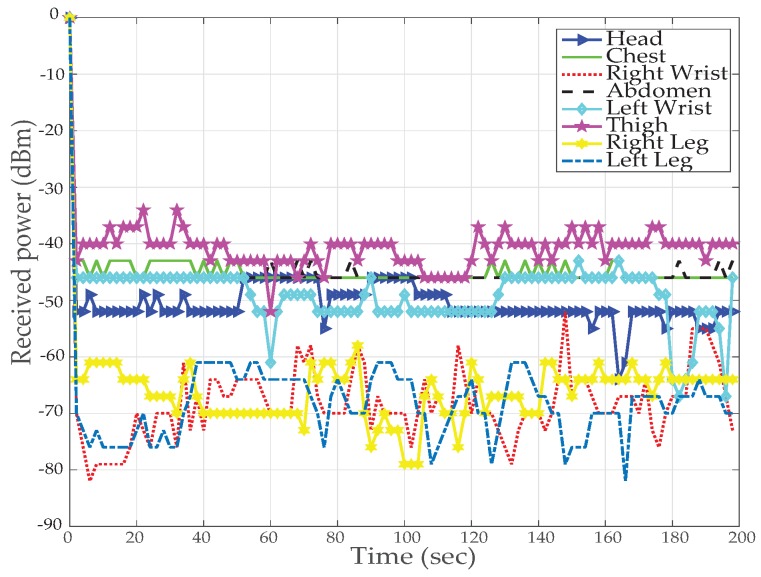
RX power using proposed dynamic control algorithm while sitting activity.

Figure 18 and Figure 19 elaborates received power results from sitting position, maintained for 3–4 min. The RSSI is reasonably good for most of the time at high transmit power (*TX*_1_). Similarly, it is also good at low transmit power. Observing the plots, it can be seen that at maximum and minimum power levels, the packet loss is almost zero. Whereas at less than minimum and medium power level packet loss is significant.

**Figure 20:**
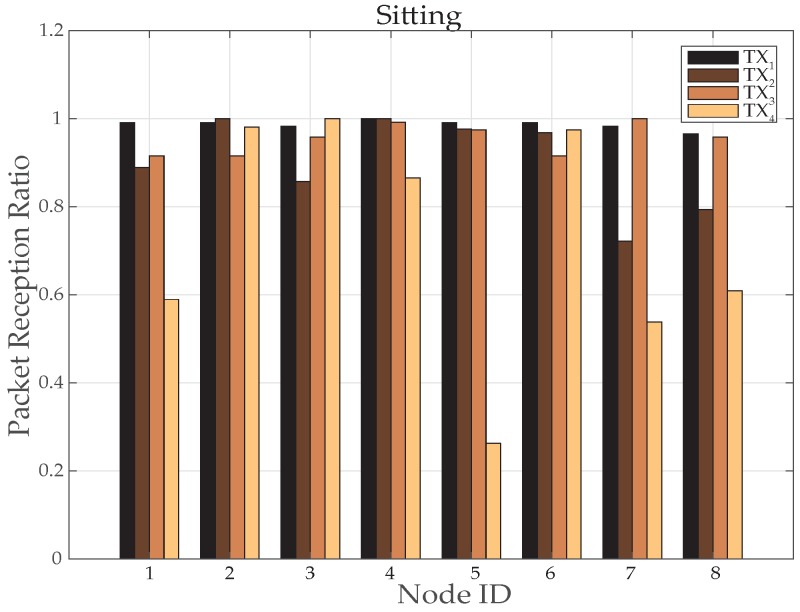
Packet Reception Ratio at various nodes while sitting activity.

In Figure 20 lesser PRR shows poor packet transmission for nodes 1, 3, 7 and 8 respectively. At the same time all of the remaining nodes (2, 4, 5 and 6) are showing highly reliable transportation with maximum packet reception. Therefore, these nodes present opportunities for energy saving and can be used with very long battery lifetime. This is what we can observe in Figure 19 that some nodes are doing transmission at lower power levels.

**Figure 21:**
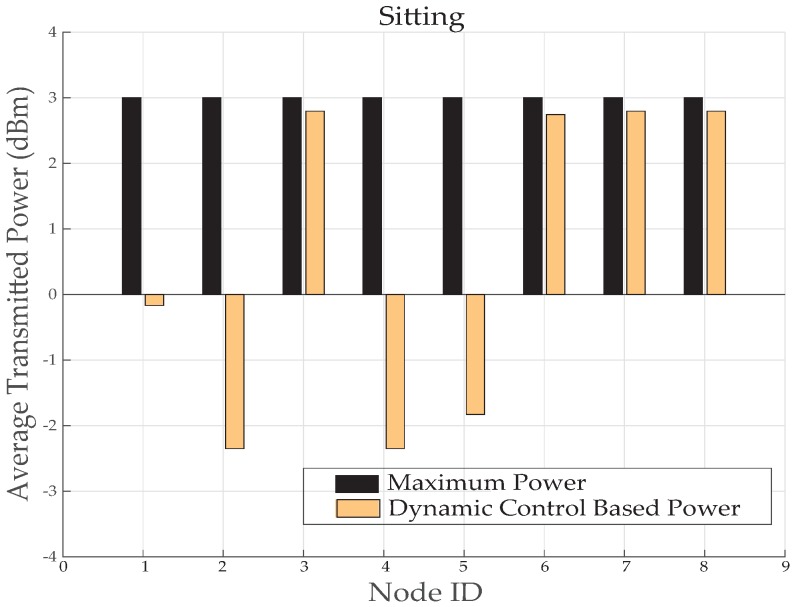
Average TX power consumption while sitting activity.

Therefore, in such situation a mechanism that automatically switches the power is highly efficient for systems with a good level of reception. An average transmit power consumption in Figure 21 also proves this technique energy efficient for sitting posture. It can be seen from Figure 21 that node ID 2, 4 and 5 has reduced power consumption up to 90%. However, node 1 has reduced to 53% and node 3, 6, 7 and 8 has reduced 3% to 5% of power.

**Figure 22:**
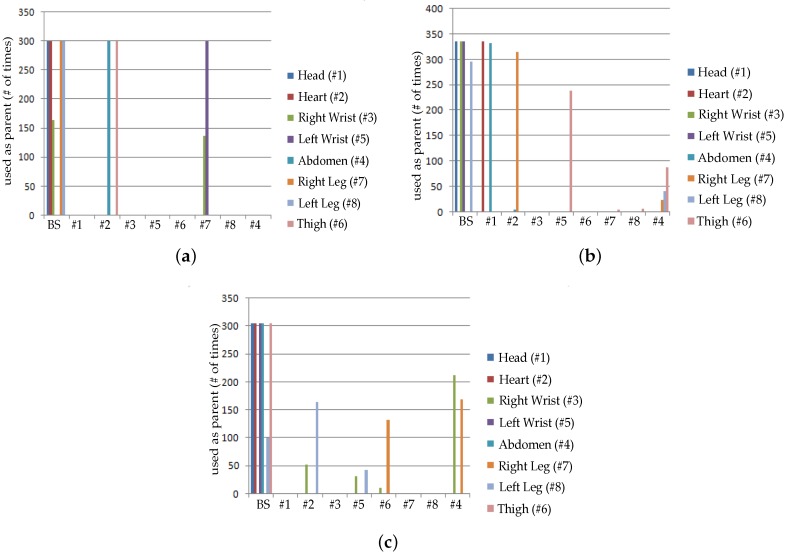
No. of Hops while sleeping activity. (**a**) At *TX*_2_; (**b**) At *TX*_3_; (**c**) At *TX*_4_.

**Figure 23:**
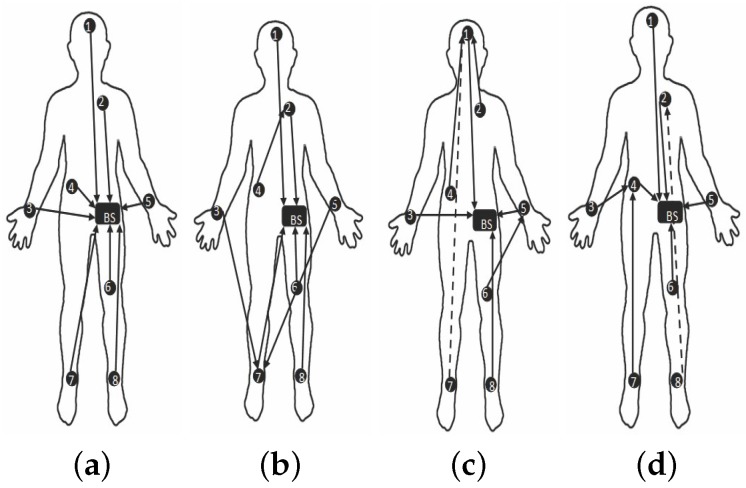
Typical network topology while sleeping activity. (**a**) At *TX*_1_; (**b**) At *TX*_2_; (**c**) At *TX*_3_; (**d**) At *TX*_4_.

Figure 22 and Figure 23 represents result for topological behavior while sleeping for 10 min. Result of *TX*_1_ (Figure 23a) shows single-hop transmission for a period of 10 min as in the *TX*_1_ of Sitting posture. It is because both body positions have less unusual movements. Furthermore, we have determined that for *TX*_2_ (Figure 23b) from the very start, some nodes are sending packets to BS using multiple hops, as shown in Figure 22a. It is also observed that for *TX*_3_ and *TX*_4_ (Figure 23c,d and Figure 22b,c), nodes have changed their routes three times to perform reliable delivery of packets. All the results obtained till now proof that low transmit power results in multi-hop topology to maintain reliable transmission of packets.

**Figure 24:**
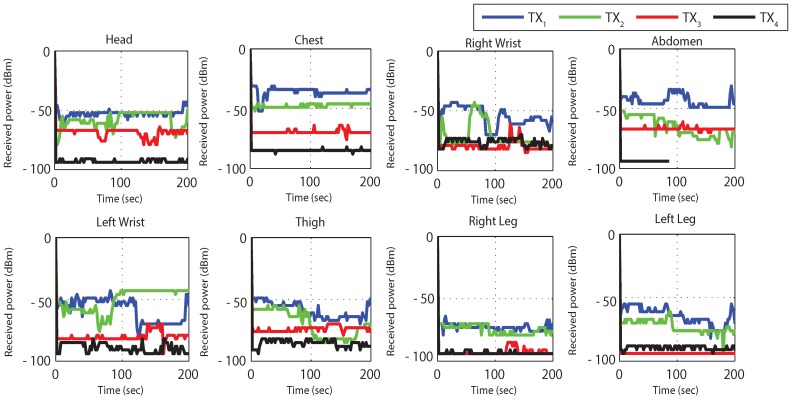
RX power based on default TX power while sleeping activity.

**Figure 25:**
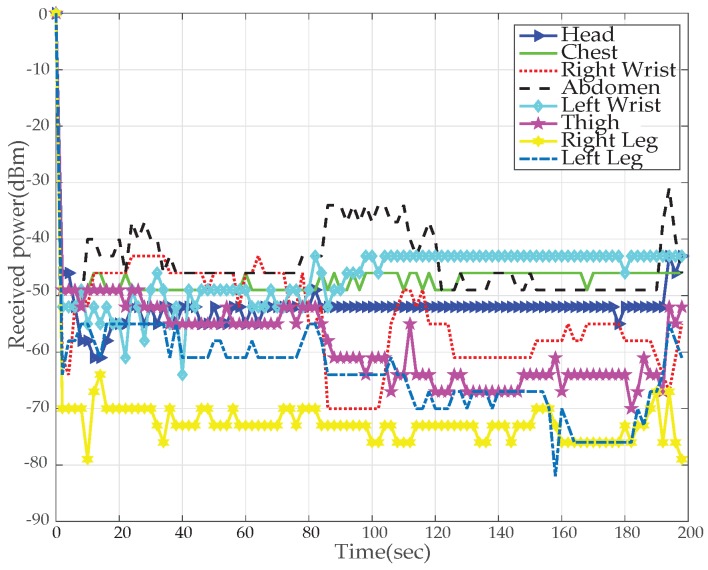
RX power using proposed dynamic control algorithm while sleeping activity.

Figure 24 and Figure 25 represents the results for RSSI reception when the patient sleeps down to rest on a bed. During this position the stability in receiving values of power is very noteworthy. The Figure 24 plots the RSSI over the entire period, at several time intervals. At maximum (*TX*_1_) and medium (*TX*_2_) transmit power levels, stable and high packet reception is achieved.

**Figure 26:**
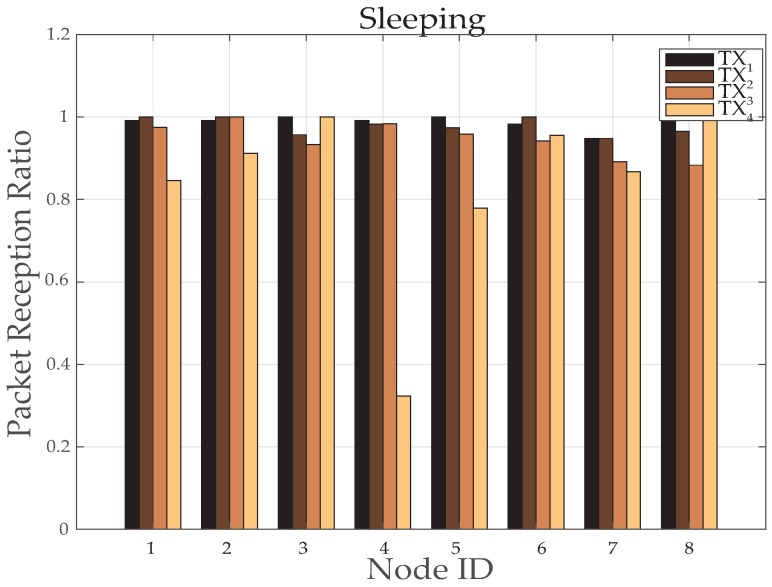
Packet Reception Ratio at various nodes while sleeping activity.

**Figure 27:**
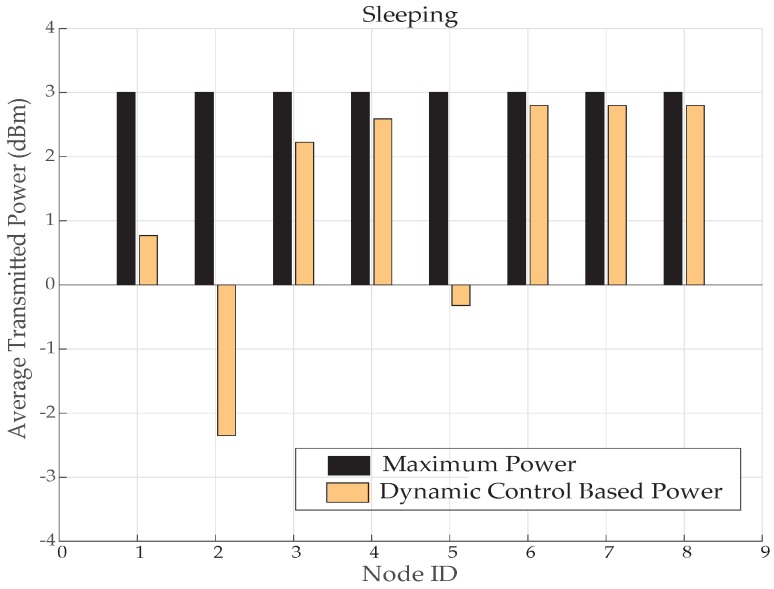
Average TX power consumption while sleeping activity.

This can also be shown from the PRR graph, in Figure 26. The energy savings at minimum transmit power is also very significant with improved packet reception by most of the nodes. Except node 7 and 8, that is, of legs, which shows very poor PRR because while sleeping nodes comes over some hidden places as happened here and *TX*_4_ proved poor idea to be utilized here. For such conditions, mechanism is designed to perform dynamic power controlling for transmitting power to maintain high reliability with less power consumption. Therefore, different levels of powers are significantly used by most of the nodes to save energy. While node 6, 7 and 8 uses a higher level of transmitting power to maintain received power with good decibel values, as shown in Figure 25. This proves in Figure 27 that nodes 2 and 5 has improved average transmitted power utilization at very good extent by saving 60% and 90% of power, respectively. However, node 1 has reduced 37% of power and node 3, 4, 6, 7 and 8 has saved 3% to 13% of energy.

The computation of all the obtained results forced us to implement an adaptive algorithm in wireless DigiAID design that decides its transmission parameters from the output values, that are back forwarded to the input and helps in increasing system lifetime by saving transmit power.

## 6. Conclusions and Future Work

The DigiAID, an open and cost-effective experimental platform for monitoring the human health conditions is compared with commercially available product called as Hexoskin. We showed the viability of DigiAID through extensive real measurements during daily activities by both male and females. The comparison between DigiAID and Hexoskin highlighted the areas where different sensors did not perform optimally. It was also revealed that wired nature of the system was also a hurdle in monitoring properly.

Furthermore, the proposed work presents the characterization of wireless DigiAID in WBSNs. Understanding the impact of body movements on packet reception and power consumption is of great importance. As far as topology is concerned, for maximum power most of the time packets utilize single-hop and usage of minimum power results in multi-hop transmission. Hopping behavior is also affected due to movements of human body. With multi-hopping if nodes are following longer routes, delay, path loss and data corruption may be experienced. It is concluded that maximum transmit power is achieved with around 100% reliability, but at the same time it reduces battery life. While minimum power gives efficient utilization of battery, but this result in poor packet reception too. This highlights the need of an energy efficient algorithm to minimize these problems. Accordingly, a dynamic power control algorithm is proposed, which controls the selection of transmit power from received PRR values. The results show an energy efficiency by reducing energy utilization with minimum of 3% and maximum of 90% while maintaining the good packet reception rate.

In future, we aim at conducting experiments that cover critical health situations, which can be effectively achieved using simulated patients. Given that field trials on real patients are hard to achieve, we are cooperating with first response groups to gain access to real patient data to validate our experimental platform. In addition, we aim at coping with inaccuracies in measurements due to either sensor or sensor placement errors as learnt from our first set of experiments. In future, we also aim to work on behavior of IEEE 802.15.6 in regular body movements.

## Figures and Tables

**Table 1:** Normal Ranges for Heart and Respiratory Rate.

Activity	Heart Rate	Respiratory Rate
	[beats/min]	[breaths/min]
Sit	60–100	12–20
Walk	60–100	18–29
Sleep	60–100	06–20

**Table 2:** Comparison between DigiAID and Hexoskin.

Activity	Platform		Good	Satisfactory	Not Satisfactory
Walking	DigiAID	RR	✓		
		HR		✓	
	Hexoskin	RR			✓
		HR	✓		
Sitting	DigiAID	RR	✓		
		HR			✓
	Hexoskin	RR		✓	
		HR	✓		
Sleeping	DigiAID	RR	✓		
		HR	✓		
	Hexoskin	RR		✓	
		HR	✓		
